# Photonic-assisted microwave signal multiplication and modulation using a silicon Mach–Zehnder modulator

**DOI:** 10.1038/srep20215

**Published:** 2016-02-02

**Authors:** Yun Long, Linjie Zhou, Jian Wang

**Affiliations:** 1 Wuhan National Laboratory for Optoelectronics, School of Optical and Electronic Information, Huazhong University of Science and Technology, Wuhan 430074, Hubei, China; 2 State Key Laboratory of Advanced Optical Communication Systems and Networks Department of Electronic Engineering, Shanghai Jiao Tong University, Shanghai 200240, P. R. China

## Abstract

Photonic generation of microwave signal is obviously attractive for many prominent advantages, such as large bandwidth, low loss, and immunity to electromagnetic interference. Based on a single integrated silicon Mach–Zehnder modulator (MZM), we propose and experimentally demonstrate a simple and compact photonic scheme to enable frequency-multiplicated microwave signal. Using the fabricated integrated MZM, we also demonstrate the feasibility of microwave amplitude-shift keying (ASK) modulation based on integrated photonic approach. In proof-of-concept experiments, 2-GHz frequency-doubled microwave signal is generated using a 1-GHz driving signal. 750-MHz/1-GHz frequency-tripled/quadrupled microwave signals are obtained with a driving signal of 250 MHz. In addition, a 50-Mb/s binary amplitude coded 1-GHz microwave signal is also successfully generated.

Photonic-assisted generation of microwave signals can find interesting applications in many microwave photonic systems, such as broad-band wireless access networks, software-defined radio, antenna remoting, phased-array antenna, and radar systems^1^. Usually, optical microwave generation is based on heterodyne techniques. Two phase-correlated optical waves with a wavelength spacing corresponding to the desired frequency of the microwave signal are generated, and then the microwave signal is obtained by beating the two optical wavelengths at a square-law photodetector (PD). Several approaches to generating two phase-correlated optical waves have been proposed and demonstrated based on free space devices^2^,^3^ and fiber devices^1^,^4^,^5^,^6^,^7^,^8^,^9^,^10^,^11^,^12^,^13^,^14^. Among these approaches, microwave frequency multiplication based on external modulation using Mach–Zehnder modulators (MZMs) has been considered an effective solution for high frequency and tunable microwave signal generation. For example, using a single MZM, a frequency-doubled microwave signal is generated in^4^ by adjusting the constant phase shift between two arms of the MZM to suppress all the even-order side bands. The beating of the ±1st-order side-bands at a PD would generate a frequency-doubled microwave signal. In addition, by introducing an optical notch filter to remove the optical carrier, a frequency-quadrupled microwave signal can also be generated^1^. The previous microwave generation technology using MZMs are based on bulked lithium niobate devices. The required driving voltage of the lithium niobate based MZM are relatively high. For high-order frequency-multiplicated signal generation using a single MZM, the required voltages of the driving microwave signals can be severalfold of the half-wave voltage, which are not easy to realize in practice.

Photonic generation of radio frequency (RF) binary digital modulation signals is another key technology in microwave photonics. Amplitude-shift keying (ASK), phase-shift keying (PSK) and frequency-shift keying (FSK) are basic modulation formats in wireless communications, which convey information by modulating the amplitude, phase or frequency of a continuous carrier wave. Traditionally, these modulated microwave signals are generated in electrical domain using digital electronic circuits^15^. Due to the electronic bottleneck, the major difficulty of this traditional technique is that the frequency of the generated signals is limited to a few GHz. An effective method to generate high frequency RF signals is to generate RF signals in the optical domain^16^,^17^,^18^. These approaches are based on spatial light modulator (SLM) technologies or fiber devices. Similar to microwave frequency mulitiplication, the use of MZM is also considered to be a competitive approach to generating binary digital modulation signals. Microwave PSK and FSK signals have been proposed and realized based on commercial MZMs^16^,^17^,^18^. However, microwave ASK signal as a fundamental digital modulation format in wireless communication has rarely been realized using MZM.

Compared to free space or fiber-based devices to generate frequency-multiplicated microwave signals or binary digital modulation signals, silicon-on-insulator (SOI) based waveguides can offer distinct advantages of increased stability and reliability, low cost, small footprints, and compatibility with other integrated optoelectronic devices^19^. In recent years, owing to the great success of silicon photonics, some passive microwave photonic devices (i.e. microwave photonic filters, microwave photonic phase shifters) based on SOI waveguides have been proposed and demonstrated showing excellent characteristics^20^,^21^,^22^,^23^,^24^,^25^,^26^,^27^.

In this paper, we propose a simple yet effective scheme to obtain frequency- multiplicated microwave signals or amplitude coded microwave signals based on a single integrated silicon MZM. In proof-of-concept experiments, 2-GHz frequency-doubled microwave signal is generated using a 1-GHz driving signal. 750-MHz/1-GHz frequency-tripled/quadrupled microwave signals are obtained with a driving signal of 250 MHz. Moreover, a 50-Mb/s binary amplitude coded 1-GHz microwave signal is also successfully generated in the experiment.

## Results

### Photonic-assisted microwave signal multiplication

**Figure 1:**
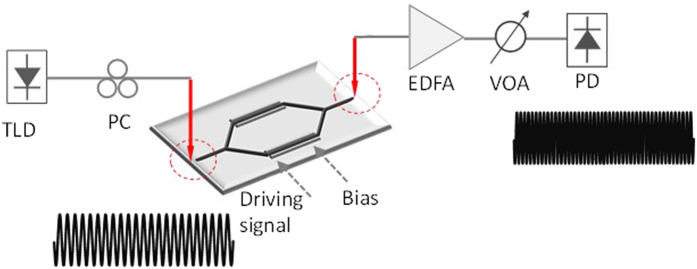
Schematic illustration of the photonic frequency-multiplicated microwave signal generation system using an integrated silicon MZM. TLD, tunable laser diode; PC, polarization controller; EDFA: erbium-doped fiber amplifier; VOA: variable optical attenuator; PD: photodetector.

Figure 1 illustrates the schematic illustration of the proposed photonic frequency-multiplicated microwave signal generation system using an integrated silicon MZM. Continuous-wave (CW) light from a tunable laser diode (TLD) is sent to the MZM. The driving signal and the DC-bias are combined by a bias-tee and applied to the MZM. The output light intensity of the MZM can be written as


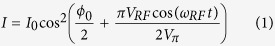


where *I*_0_ is the intensity of the input optical carrier. *φ*_*0*_ = *πV*_*b*_* /V*_*π*_is the constant phase difference between the two arms determined by the constant DC-bias voltage *V*_*b*_. *V*_*π*_ is the half-wave voltage of the MZM. *V*_*RF*_ and *ω*_*RF*_ are the amplitude and angular frequency of the applied electrical drive voltage, respectively. Based on the Jacobi–Anger expansions, Eq. (1) can be expanded to be


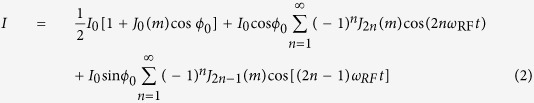


where *m* = *πV*_*RF*_*/V*_*π*_ is the modulation indice of the MZM. *J*_*n*_ is the *n*th order Bessel function of the first kind.

It follows from Eq. (2) that if the modulator is biased at *V*_*π*_, the odd components will be suppressed. Ignoring the high order harmonic wave, a frequency-doubled microwave signal is obtained. In addition, by adjusting the driving microwave signal amplitude to let *J*_*2*_(*m*) = 0, the quadruple response will dominant the output, and a frequency-quadrupled signal can be obtained. Similarly, a frequency-tripled signal is generated by suppressing the even components and *J*_*1*_(*m*) term of the signal.

**Figure 2:**
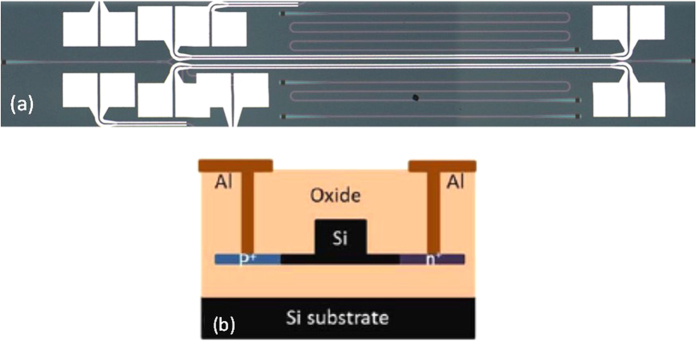
(**a**) Microscope image of the fabricated integrated MZM. (**b**) Cross-sectional view schematics of the doped waveguide.

Figure 2(a) shows the optical microscope image of the fabricated device. The waveguides are 220 nm high with a 60 nm slab. The width of the waveguide is 500 nm. The active arm length is 1.8 mm while the arm length difference between two arms are 494 μm. Figure 2(b) shows the cross-sectional view schematics of the doped waveguide.The n and p doping regions are 4 μm wide, and each has a separation of 0.6 μm from the waveguide sidewall. The doping concentration is about 10^20^ cm^−3^. Vertical grating couplers are used to couple light in/out of the silicon chip and ensure operation with transverse electric (TE) polarized light. The grating coupling loss is about 7 dB per facet.

**Figure 3:**
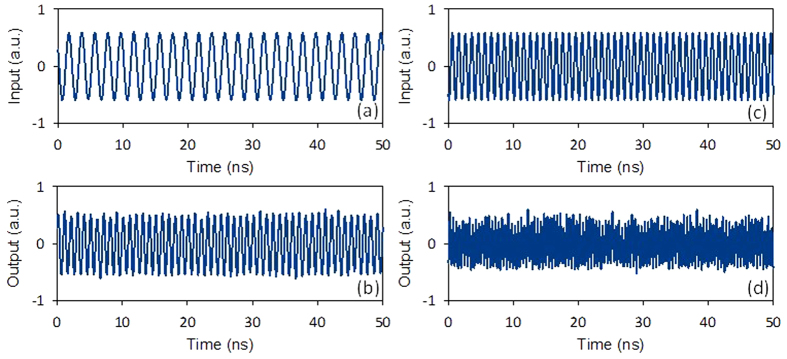
Waveforms of (**a**) the input 500-MHz driving signal, (**b**) the generated 1-GHz frequency-doubled signal, (**c**) the input 1-GHz driving signal, and (**d**) the generated 2-GHz frequency-doubled signal.

**Figure 4:**
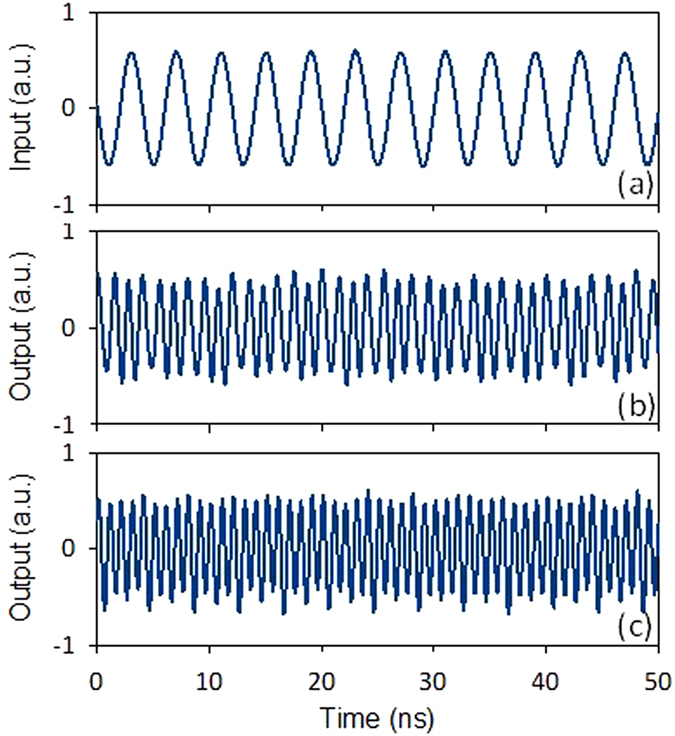
Waveforms of (**a**) the input 250-MHz driving signal, (**b**) the generated 750-MHz frequency-tripled signal, and (**c**) the 1-GHz frequency-quadrupled signal.

We first investigate the generation of frequency-doubled microwave signal. Figure 3(a,b) show the waveforms of the original 500-MHz driving signal and the generated 1-GHz signal. Figure 3(c,d) show the obtained 2-GHz signal when the driving signal frequency is 1 GHz. Figure 4 illustrates the results of the generation of frequency-tripled and frequency- quadrupled signal with a driving signal of 250 MHz. The imperfections of the waveforms come from the influence of incompletely suppressed high-order components.

### Photonic-assisted microwave ASK modulation

**Figure 5:**
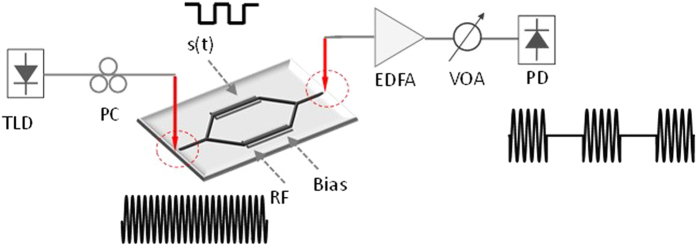
Schematic illustration of the proposed photonic microwave ASK signal generator.

Using the fabricated integrated MZM, we also demonstrate the feasibility of microwave ASK modulation using integrated photonic approach. Figure 5 illustrates the schematic illustration of the proposed photonic microwave ASK signal modulation system using an integrated silicon MZM. The microwave carrier signal and the binary coding signal *s* (*t*) are applied to the two RF ports of the MZM, respectively. The AC term of the detected signal at the output of the PD can be written as


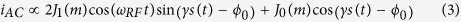


where *ω*_*RF*_, *m, V*_*RF,*_ *V*_*π*_ and *φ*_*0*_are defined as previously mentioned. *γ* = *πV*_*s*_*/V*_*π*_is the modulation index in the arm where the binary coding signal is applied. Assuming *φ*_*0*_ = 0, Eq. (3) can be simplified as


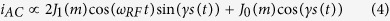


The second term in Eq. (4) is located in the baseband which can be easily eliminated by an electric filter. Assuming *γ* = *π/*2, the amplitude of the obtained signal is


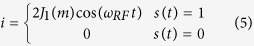


As can be seen, the amplitude of the carrier is “1” with bit “1”, and “0” with bit “0”. Therefore, two-level microwave ASK signal is generated.

**Figure 6:**
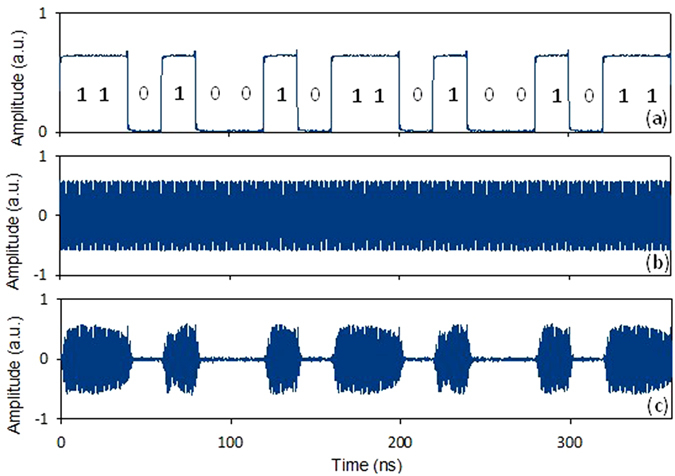
Waveforms of (**a**) the original 50-Mb/s baseband signal with a pattern of “110100101101001011”, (**b**) original 1-GHz microwave carrier signal, and (**c**) the output microwave ASK signal.

Figure 6(a) shows the waveforms of the original 50-Mb/s baseband signal with a pattern of “110100101101001011”. The original 1-GHz microwave carrier is shown in Fig. 3(b). Figure 3(c) shows the modulated microwave ASK signal. The obtained results shown in Figs 3, 4 and 6 indicate the successful implementation of photonic-assisted microwave signal multiplication and modulation using a compact silicon MZM.

## Discussion

In summary, a simple and compact photonic scheme to obtain frequency-multiplicated microwave signal has been proposed and experimentally demonstrated based on a single integrated silicon MZM. In a proof-of-concept experiment, 2-GHz frequency-doubled microwave signal is generated using a 1-GHz driving signal. Also, 750-MHz/1-GHz frequency-tripled/quadrupled microwave signals are obtained with a driving signal of 250 MHz. Furthermore, using the fabricated integrated silicon MZM, the feasibility of microwave ASK modulation has also been demonstrated. A 50-Mb/s binary amplitude coded 1-GHz microwave signal is successfully generated. The frequency of the generated microwave signals is limited by the operation bandwidth of the pin-type MZM. For the MZM design, 2 design parameters, i.e. doping structures and the length of the MZM, could influence the system performance. Using pin-type doping might reduce the requirement of input signal amplitude. However, realizing high frequency multiplication and ASK signal modulation is a great challenge. By using pn-type doping, the bandwidth could be effectively improved^28^, while the high requirement of input signal amplitude is difficult to satisfy. Extending the length of the arms of MZM might alleviate the requirement of input signal amplitude, but could suffer from extra loss. To enable high frequency microwave signal multiplication and ASK signal generation, a high bandwidth, low half-wave voltage, and low loss MZM is needed, which might be realized by employing some other doping structures such as zigzag structure to increase the modulation efficiency^29^. With future improvement, one would also expect to see high frequency microwave signal generation/modulation and other photonic-assisted microwave signal processing applications exploiting compact integrated silicon photonic devices.

## Method

### Devices fabrication

We employ a single MZM to generate various microwave signals. The designed MZM is fabricated on an SOI wafer. The thickness of the top silicon and the buried oxide layer of the SOI wafer are 220 nm and 2 mm, respectively. A deep ultra-violet (DUV) photolithography process is employed to define the waveguide patterns, followed by anisotropic dry etch of silicon. Boron and phosphorus ion implantations were performed to form the p -type and n-type doped regions. Finally, contact holes were etched and aluminum was deposited to form the metal connection. We use vertical grating coupling method to couple the fiber and the silicon chip. The whole fabrication process is done using CMOS compatible processes.

## Additional Information

**How to cite this article**: Long, Y. *et al*. Photonic-assisted microwave signal multiplication and modulation using a silicon Mach–Zehnder modulator. *Sci. Rep.* **6**, 20215; doi: 10.1038/srep20215 (2016).
